# The prevalence and distribution of the flexor carpi radialis brevis muscle in the Turkish population

**DOI:** 10.1038/s41598-021-04445-8

**Published:** 2022-01-10

**Authors:** R. F. Akkoc, F. Aksu, E. Emre, M. Ogeturk

**Affiliations:** Department of Anatomy, Faculty of Medicine, Firat University, 23119 Elazig, Turkey

**Keywords:** Anatomy, Muscle

## Abstract

The flexor carpi radialis brevis (FCRB) is a very rare anomalous muscle that is usually asymptomatic but may cause various pathologies, such as radial-sided wrist pain. The aim of this study was to determine the prevalence of FCRB in the Turkish population, its location, and sex differences. Forearm, wrist, and hand magnetic resonance images of 849 individuals aged 18–65 years were retrospectively evaluated in this study. The survey found an FCRB prevalence of 4%, with a prevalence of 3.6% among women and of 4.7% among men. However, the difference between the sexes was not statistically significant (p = 0.629). The origin of all 34 FCRBs identified was the distal third of the anterior aspect of the radius; the insertion site of 28 was the second metacarpal bone, whereas that of the remaining 6 was the os trapezium. In conclusion, the data of this study report the prevalence of FCRB for the first time in the Turkish population, which will contribute to radiological and surgical evaluations in the region and help in early and accurate diagnosis of various pathological conditions that may be caused by FCRB.

## Introduction

The flexor carpi radialis brevis (FCRB) is a rare muscle in the distal forearm and wrist^1^. It was first described as *radio carpien* by Fano in 1851^2^ and named *flexor carpi radialis brevis vel profundus* by Wood in 1867^3^. Although the FCRB has been observed in a few cadaveric dissection studies since the nineteenth century, its presence in a living person was first reported in 2006^4–6^.

The prevalence of FCRB has been reported to be between 2 and 8% in studies conducted in various populations^1,5,7^. The origin of the FCRB is the distal third of the anterior aspect of the radius or the anterolateral part of the radius located between the origin of the flexor pollicis longus (FPL) muscle and the insertion of the pronator quadratus (PQ) muscle. The insertion site of FCRB is usually the base of the second or third metacarpal bones but is sometimes the base of the fourth metacarpal bone and the radial side of os scaphoideum, os trapezium, os trapezoideum, or os capitatum^4,8,9^.

FCRB, which has a very low prevalence, rarely causes clinical symptoms. When symptomatic, radial-sided wrist pain and sometimes wrist edema occur in the area that matches the location of the FCRB. Radial-sided wrist pain can pose a diagnostic challenge for radiologists and other clinicians, as it may be due to bone, cartilage, ligament, tendon, muscle, or neurovascular bundle damage. This symptom has a long list of differential diagnoses, including tendinopathy, tendon tear, ligamentum damage or instability, acute fracture, degenerative arthritis, and ganglion cyst^10^.

This study aimed to determine FCRB, an extremely rare anatomical variation, characteristics of prevalence, distribution, origin, insertion, and sex in the Turkish population. To our knowledge, there is no study in the literature on the prevalence of FCRB in this population to date. In addition, the FCRB has clinical importance due to its proximity to the standard approach for distal radius fracture^6,8,11,12^. Therefore, understanding the importance of anatomical variations, abnormal muscle presence, and different origins and insertions of muscles by both radiologists and surgeons and providing awareness of the existence of these differences in surgical and medical treatments was another aim of the study.

## Methods

### Ethics statement

The use of imaging data in this study was reviewed and approved by the Fırat University Non-Interventional Research Ethics Committee (dated 28.03.2019, and numbered 05 and 09). The study was conducted in accordance with the principles of the Declaration of Helsinki. All patients provided written informed consent.

### Study population

**Figure 1:**
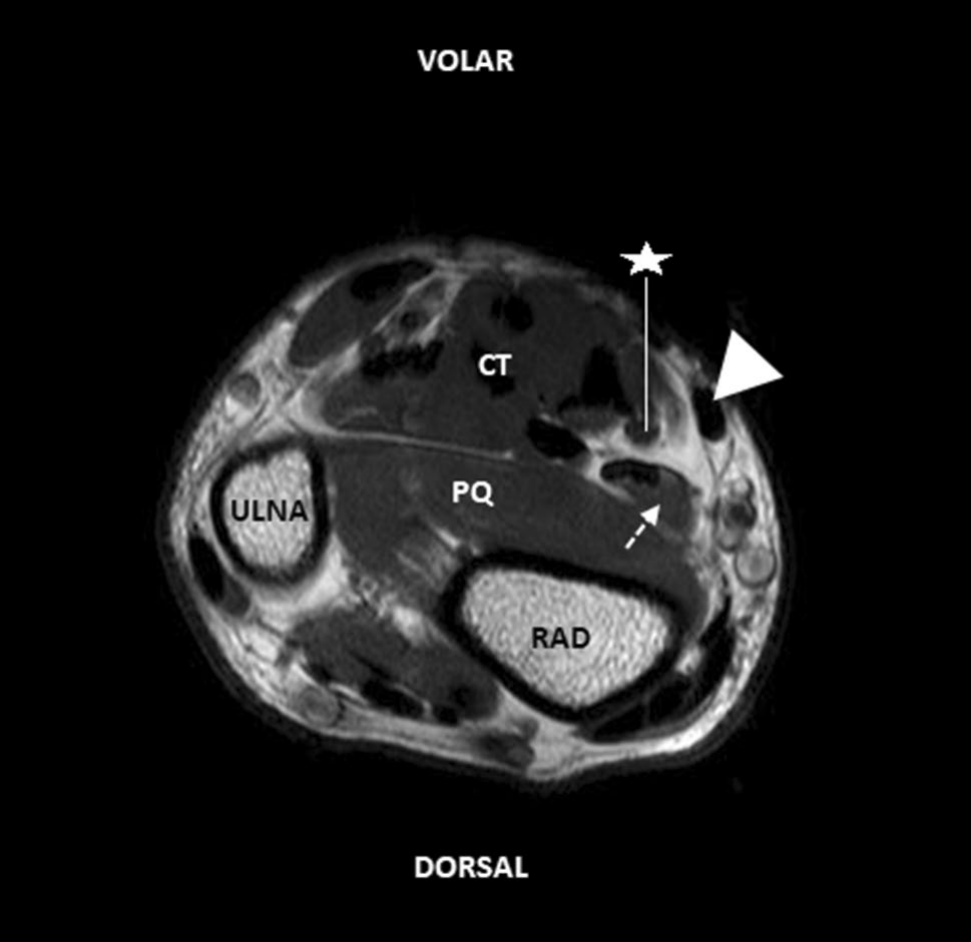
Axial T1W TSE MR image of the wrist; m. flexor carpi radialis tendon (arrowhead), m. flexor pollicis longus (dotted arrow), carpal tunnel contents (CT), m. pronator quadratus (PQ), radius (RAD), n. medianus (star).

Forearm, wrist, and hand magnetic resonance (MR) images of a total of 849 patients, 507 women and 342 men, aged between 18–65 years, who applied to Fırat University Hospital between April 01, 2014, and March 31, 2019, were evaluated retrospectively (Fig. 1).

### MR image examination

MR images with the presence of fractures or fissures in the radius, ulna, carpal, or metacarpal bones, those with operation scars in the same regions and wrist joint pathology, and those with poor image quality were excluded.

The presence, localization, origin, and insertion of the FCRB on MR images were analyzed, and the data were recorded. T1W TSE sequences of axial and coronal slices of the evaluated MR images were used.

MR images being used in the study are the images of the patients who applied to the Fırat University Hospital and were shot through Philips Ingenia (Netherlands) MR device, which has 1.5 Tesla magnetic field strength. Enlil PACS System-2.5 (Enlil PACS Viewer, Eroglu Software Inc., Eskisehir, Turkey) is used during MR evaluations at Fırat University Hospital. Physical parameters are TE: 15, TR: 651.5 for axial slices, and TE: 22, TR: 450 for coronal slices.

### Statistical analysis

SPSS (IBM Corp., Version 22.0) was used for statistical analysis of the data. The Chi-square test was applied for intergroup comparisons. Categorical measurements are given as numbers and percentages and continuous measurements as the mean and standard deviation (median and minimum–maximum where necessary). For statistical significance, a level of 0.05 was accepted in all tests.

## Results

**Figure 2:**
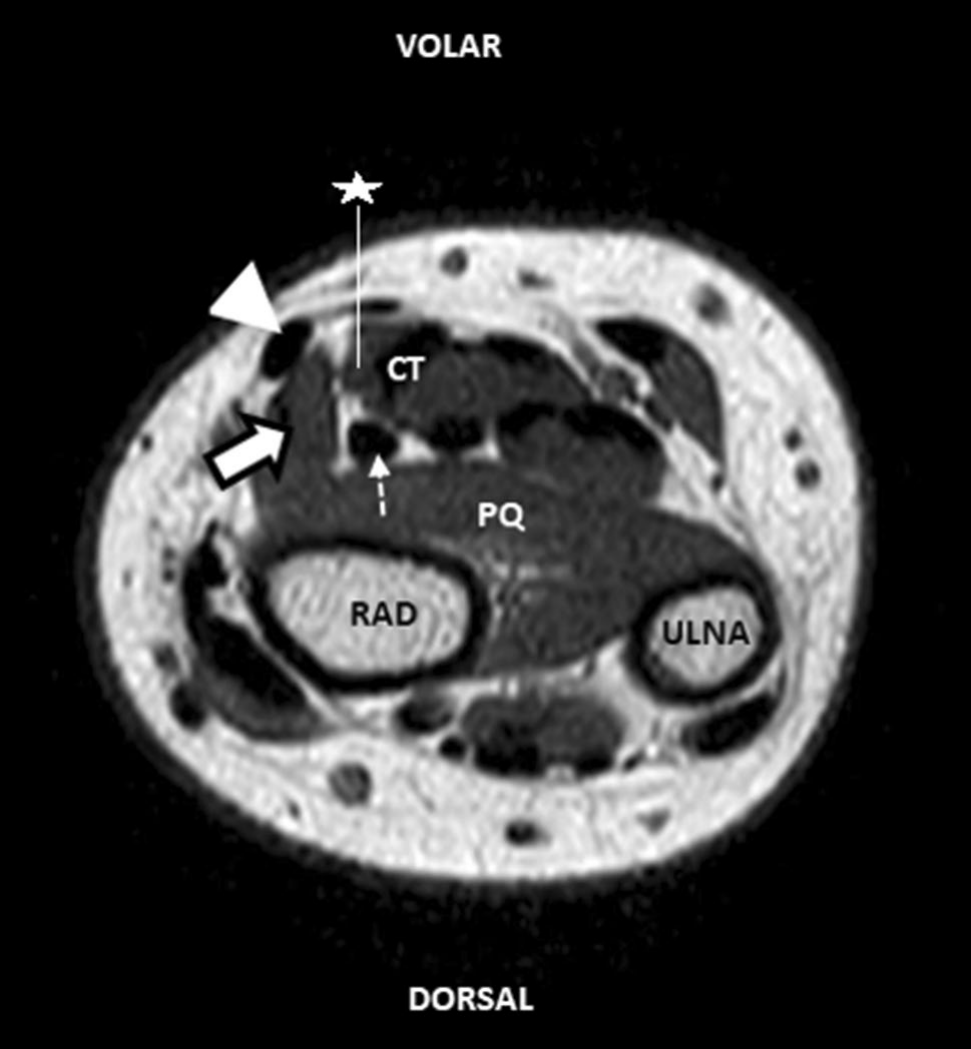
Axial T1W TSE MR image of the wrist; m. flexor carpi radialis tendon (arrowhead), m. flexor carpi radialis brevis (arrow), m. flexor pollicis longus (dotted arrow), carpal tunnel contents (CT), m. pronator quadratus (PQ), radius (RAD), n. medianus (star).

The mean age of the 849 people included in the study was 36.95 ± 13. Of the 849 MR images analyzed retrospectively, 507 (59.7%) were from females and 342 (40.3%) from males. The prevalence of the FCRB was determined to be 18 (3.6%) of 507 females and 16 (4.7%) of 342 males, for 34 (4%) of 849 individuals in total (Fig. 2). There was no statistically significant difference in the prevalence of the FCRB between the sexes (p = 0.629). Of the 849 MR images examined, 466 were images of the right upper extremity; 383 were of the left upper extremities. The right-side FCRB prevalence was 18 (3.9%) of 466 people; the left-side FCRB prevalence was 16 (4.2%) of 383 people. Of 466 people with right upper-extremity images, 275 (59%) were female and 191 (41%) male (Table 1); of 383 people whose left upper-extremity image was evaluated retrospectively, 232 (60.5%) were female and 151 (39.5%) male (Table 2).

**Table 1:** FCRB distribution on right upper-extremity MR images [n (%)].

	No	Yes	Total
Female	266 (96.7)	9 (3.3)	275
Male	182 (95.3)	9 (4.7)	191
Total	448 (96.1)	18 (3.9)	466

*FCRB* musculus flexor carpi radialis brevis, *MR* magnetic resonance.

**Table 2:** FCRB distribution on left upper-extremity MR images [n (%)].

	No	Yes	Total
Female	223 (96.1)	9 (3.9)	232
Male	144 (95.4)	7 (4.6)	151
Total	367 (95.8)	16 (4.2)	383

*FCRB* musculus flexor carpi radialis brevis, *MR* magnetic resonance.

**Figure 3:**
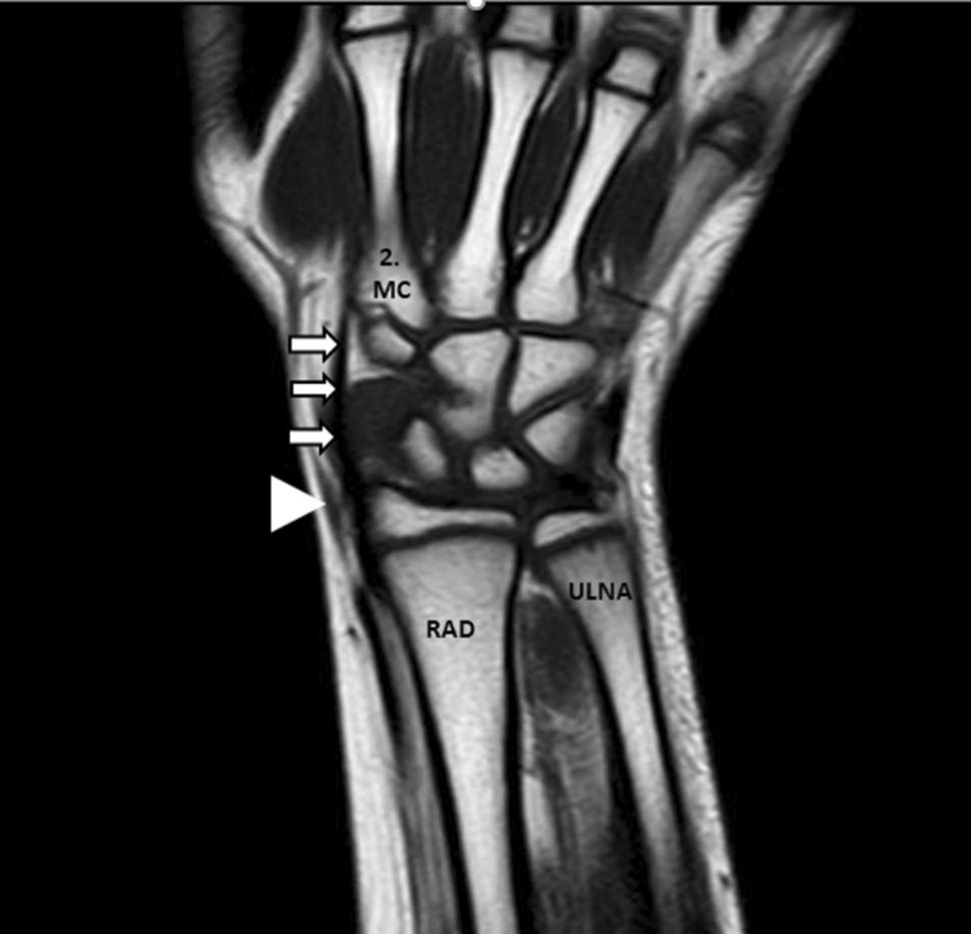
Coronal T1W TSE MR image of the wrist; m. flexor carpi radialis tendon (arrowhead), m. flexor carpi radialis brevis tendon (arrow), radius (RAD), os metacarpale (MC).

**Figure 4:**
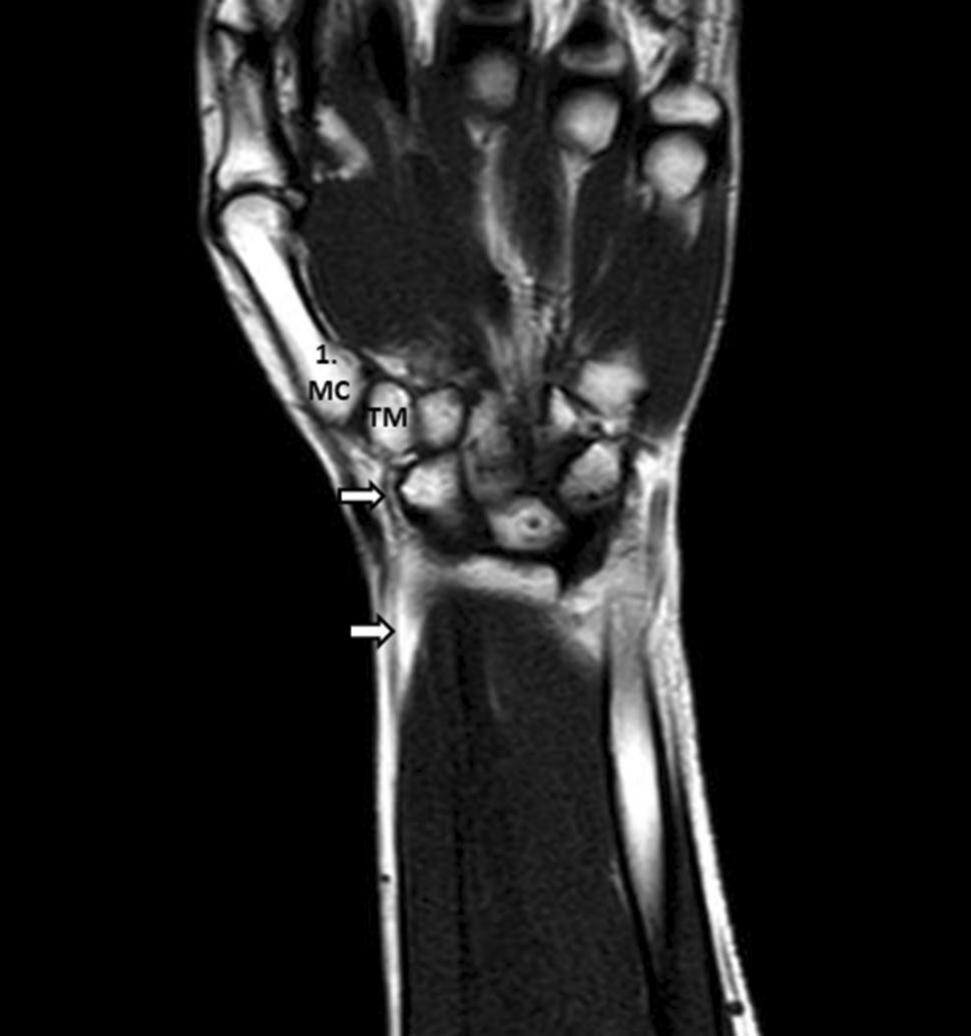
Coronal T1W TSE MR image of the wrist; m. flexor carpi radialis tendon (arrow), os metacarpale (MC), os trapezium (TM).

The origin of 34 FCRBs detected on the MR images examined was the distal third of the anterior surface of the radius. In addition, the insertion of FCRB was the second metacarpal bone in 82.4% (Fig. 3) and the os trapezium in 17.6% (Fig. 4). The distribution of FCRB insertions by sex is presented in Table 3.

**Table 3:** Distribution of FCRB insertion sites [n (%)].

	Second Metacarpal	Os Trapezium	Total
Female	16 (88.9)	2 (11.1)	18
Male	12 (75)	4 (25)	16
Total	28 (82.4)	6 (17.6)	34

*FCRB* musculus flexor carpi radialis brevis.

## Discussion

FCRB is rarely observed in the distal forearm and wrist^1^. Innervation of the FCRB is provided by the anterior interosseous nerve, and feeding is supplied by the radial artery^5,13,14^. The FCRB is a striated skeletal muscle and plays a role in voluntary wrist flexion^15^.

Overall, there are very few studies investigating the prevalence of the FCRB in the literature. A few studies of the FCRB, in which the prevalence varies between 2 and 8%, have been conducted via cadaver dissections, surgical procedures for distal radius fractures, and MRI scans^1,12,14,16^. Mantovani et al.^12^ reported a 3.5% presence of the FCRB in 172 patients with distal radius fractures, whereas Lee et al.^1^ reported a rate of 2.8% among 71 cases of distal radius fracture. In other studies, Yoshida et al.^16^ found a presence of the FCRB of 1.7% among 225 cadavers, and Mimura et al.^14^ calculated a ratio of 1.6% based on the MR images of 379 people with carpal tunnel syndrome. The presence of the FCRB was 4% according to MRI images of the 849 subjects examined in this study.

Mimura et al.^14^ reported that 6 FCRBs, they identified in 129 males and 250 females with carpal tunnel syndrome, and all belonged to female patients; four of them were on the right side, and two were on the left side. Mantovani et al.^12^, on the other hand, reported that of the 6 FCRBs they detected in 100 males and 72 females with distal radius fracture, four were on the right side and two were on the left side; three were found in male patients and three in female patients, with no correlation between the FCRB presence and sex. Of the 34 FCRBs identified in the present study, 18 were in females and 16 in males, 18 of them were on the right side and 16 on the left side. In addition, there was no statistically significant difference between the sexes and the presence of the FCRB, in line with the report of Mantovani et al.^12^.

In other studies, FCRB insertion has often been reported as the base of the second or third metacarpal bone. Furthermore, it has been found that FCRB rarely terminates in the base of the fourth metacarpal bone, os scaphoideum, os trapezium, os trapezoideum, or os capitatum^4,8,9^. In our study, similar to the literature, 28 (82.3%) of 34 FCRB insertions terminated in the second metacarpal; 6 (17.7%) terminated in the os trapezium.

**Figure 5:**
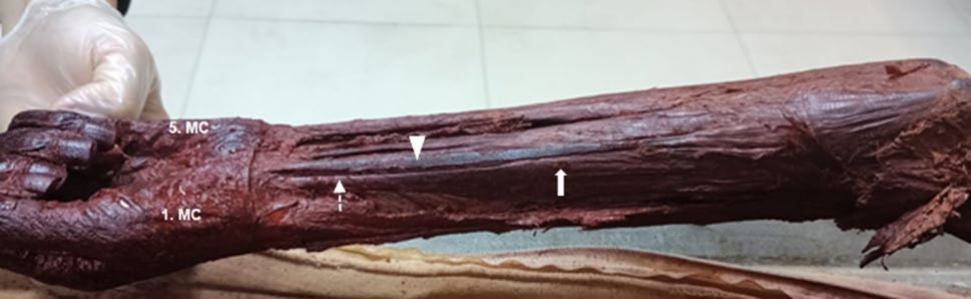
An image of a cadaver without the flexor carpi radialis brevis muscle; m. flexor carpi radialis (arrow), m. flexor carpi radialis tendon (dotted arrow), m. palmaris longus tendon (arrowhead), os metacarpale (MC).

Symptoms caused by FCRB are related to its location and function^17–19^. Radial-sided pain of the wrist, interosseous anterior compression, inflammation due to overuse of the muscle where it attaches to the radius, and FCRB tenosynovitis are some of the disorders that may occur^19,20^. In distal forearm surgeries, especially in the approach for distal radius fracture, the flexor carpi radialis (FCR) muscle is a location determinant because of its strong and thick structure, rare anatomical variations, and superficial palpability, and such operations are planned according to the FCR^21,22^ (Fig. 5). In addition to some symptoms that may be caused by an FCRB located close to the FCR, another important clinical aspect is related to distal radius fracture surgery with the standard approach site. In this type of operation, surgeons are more likely to encounter the FCRB due to its location^1^.

The prevalence of the FCRB in the Turkish population was found to be 4%. In addition, while the origin of the FCRB was the distal third of the anterior aspect of the radius, its insertion was usually the second metacarpal bone and, more rarely, the os trapezium. Clinicians, especially orthopedists, may encounter an FCRB during radiological evaluations or surgical interventions. Clinicians' awareness of the presence of the FCRB and its anatomical location (origin and insertion) may reduce the risk of FCRB-induced complications in surgical procedures. We also suggest that knowing that the FCRB causes various pathologies, such as radial-sided wrist pain and interosseous anterior nerve compression, will contribute to early and accurate diagnosis.

Clinicians, especially orthopedists, may encounter FCRB during radiological evaluations or surgical interventions. Clinicians' awareness of the presence of FCRB and its anatomical location (origin and insertion) may reduce the risk of FCRB-induced complications in surgical procedures. In addition, we think that knowing that FCRB causes various pathologies such as radial wrist pain and n. interosseous anterior compression will contribute to an early and accurate diagnosis.
