# Addendum: The prevalence and distribution of the flexor carpi radialis brevis muscle in the Turkish population

**DOI:** 10.1038/s41598-022-18623-9

**Published:** 2022-09-08

**Authors:** R. F. Akkoc, F. Aksu, E. Emre, M. Ogeturk

**Affiliations:** Department of Anatomy, Faculty of Medicine, Firat University, 23119 Elazig, Turkey

Addendum to: *Scientific Reports* https://doi.org/10.1038/s41598-021-04445-8, published online 10 January 2022

Following publication, concerns have been raised that annotations in some figures depicting magnetic resonance images mask important features. A supplementary information file containing un-annotated images corresponding to Figures 1-4 is linked to this notice.

## Supplementary Information

Supplementary Information.

