# Supplementary material for: Addendum: The prevalence and distribution of the flexor carpi radialis brevis muscle in the Turkish population
Source: Sci Rep. 2022 Sep 8;12:15226. doi: 10.1038/s41598-022-18623-9 (PMC9458718; doi:10.1038/s41598-022-18623-9)
Supplement: Supplementary file 1 — Supplementary Information. [file 41598_2022_18623_MOESM1_ESM.docx]

**Addendum: The prevalence and distribution of the flexor carpi radialis brevis muscle in the Turkish population**

R. F. Akkoc^1 *^, F. Aksu^1^, E. Emre^1^ & M. Ogeturk^1^

*^1^Department of Anatomy, Faculty of Medicine, Firat University, 23119, Elazig, Turkey*

^*^ **Corresponding author** Ramazan Fazil Akkoc, Department of Anatomy, Faculty of Medicine, Firat University, Elazig, Turkey, e-mail: ramazan_fazil@hotmail.com


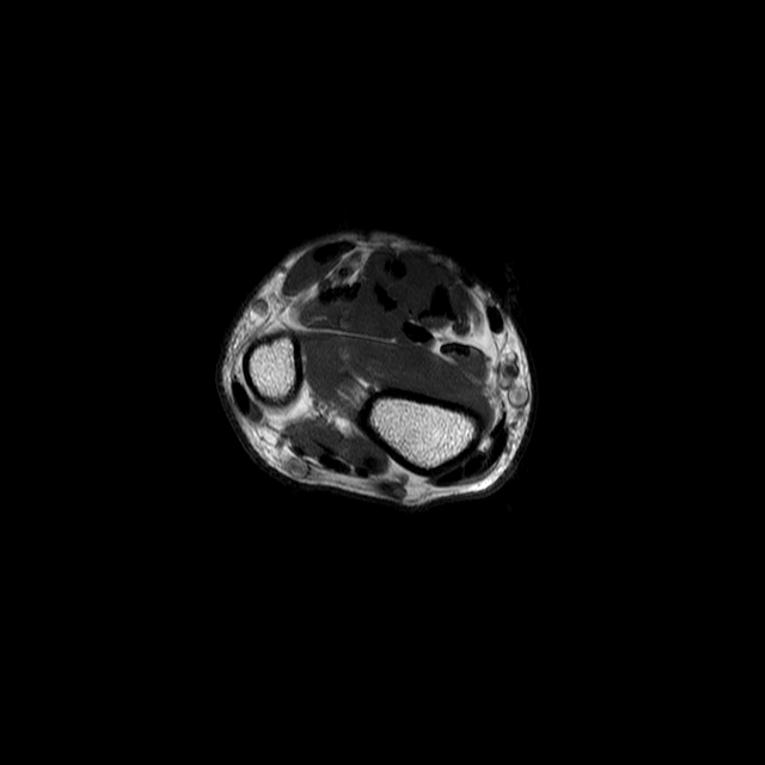


**Figure 1.** Un-annotated image corresponding to Figure 1. Axial T1W TSE MR image of the wrist

**
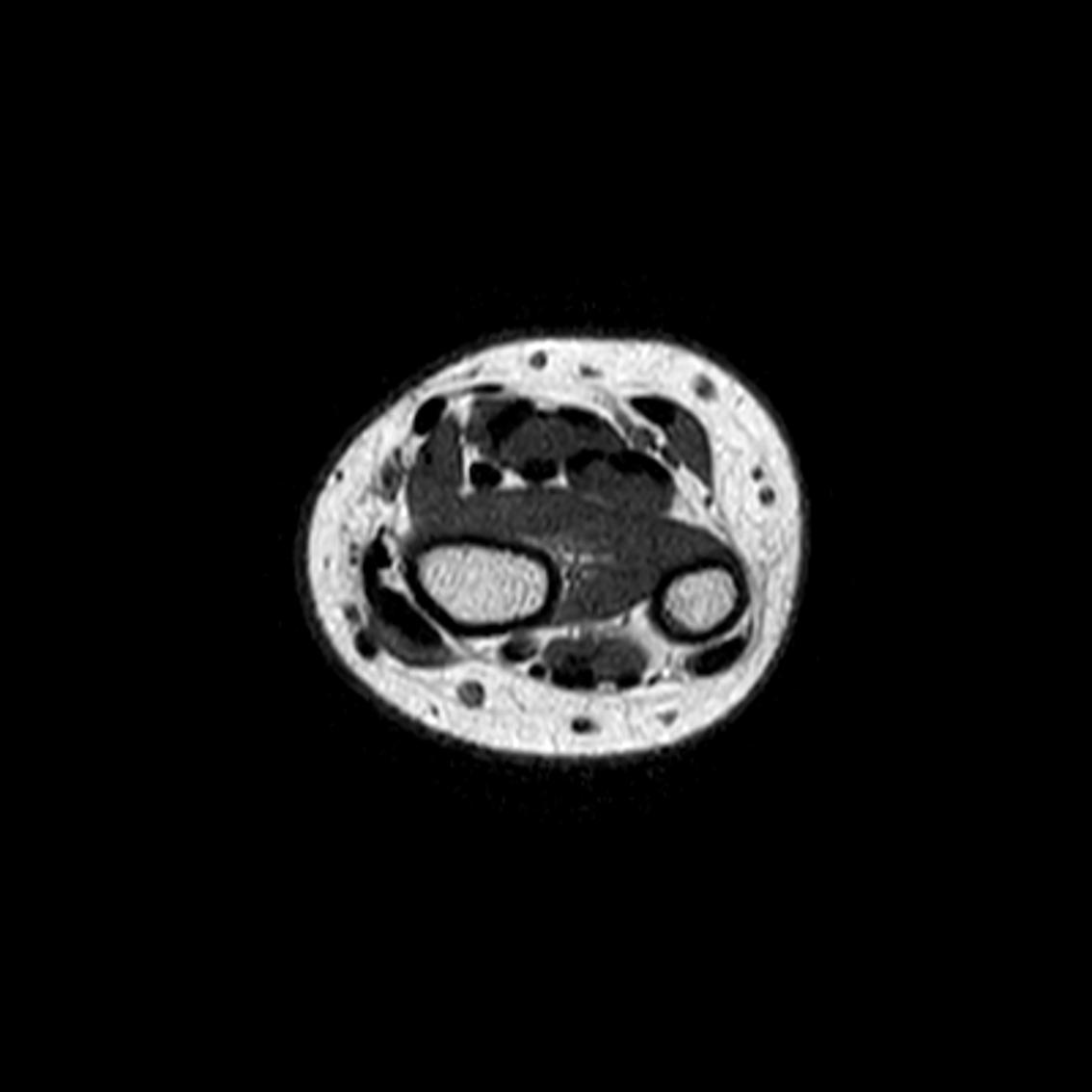
**

**Figure 2.** Un-annotated image corresponding to Figure 2. Axial T1W TSE MR image of the wrist


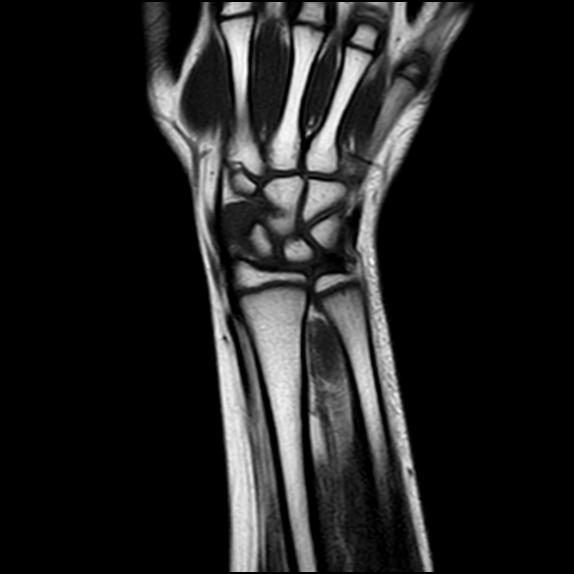


**Figure 3.** Un-annotated image corresponding to Figure 3. Coronal T1W TSE MR image of the wrist


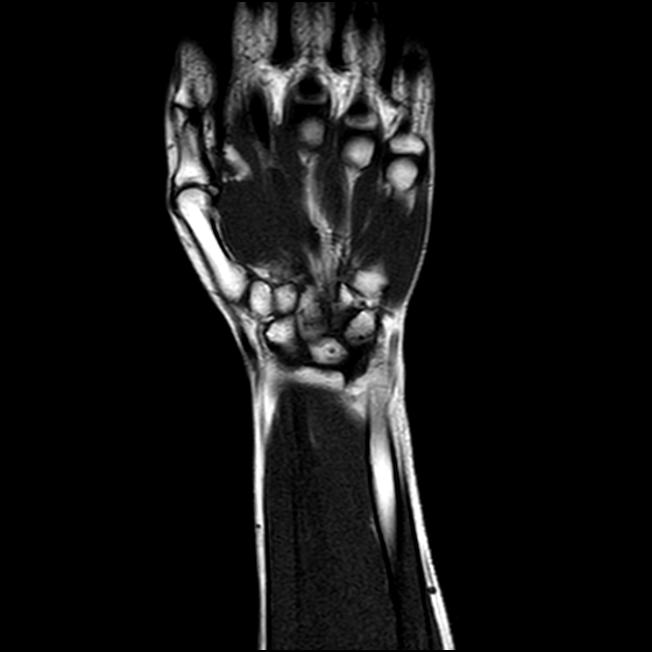


**Figure 4.** Un-annotated image corresponding to Figure 4. Coronal T1W TSE MR image of the wrist
